# MicroRNA-483-5p Predicts Poor Prognosis and Promotes Cancer Metastasis by Targeting *EGR3* in Nasopharyngeal Carcinoma

**DOI:** 10.3389/fonc.2021.720835

**Published:** 2021-10-15

**Authors:** Xi-Zhao Li, Yi-Jun Tu, Ting Zhou, Jiang-Bo Zhang, Ruo-Wen Xiao, Da-Wei Yang, Pei-Fen Zhang, Peng-Tao You, Xiao-Hui Zheng

**Affiliations:** ^1^ State Key Laboratory of Oncology in South China, Collaborative Innovation Center for Cancer Medicine, Guangdong Key Laboratory of Nasopharyngeal Carcinoma Diagnosis and Therapy, Sun Yat-sen University Cancer Center, Guangzhou, China; ^2^ Hubei Key Laboratory of Resources and Chemistry of Chinese Medicine, College of Pharmacy, Hubei University of Chinese Medicine, Wuhan, China

**Keywords:** nasopharyngeal carcinoma, *EGR3*, miR-483-5p, prognosis, metastasis

## Abstract

**Background:**

MicroRNAs, as small non-coding RNAs, play an important role in tumorigenesis. MiR-483-5p was found to have a significant increase as a diagnostic biomarker of nasopharyngeal carcinoma (NPC), not only in plasma from NPC patients but also in tumor cell lines and biopsy tissues in our previous study. However, its function and mechanism in NPC are still unclear.

**Methods:**

Tissue microarray including 178 primary NPC and 35 adjacent non-cancerous nasopharyngeal mucosal tissues was used to further validate the overexpression of miR-483-5p. Wound healing and invasion assays were conducted to verify its biological function. RNA sequencing (RNA-seq) and dual-luciferase reporter assay was performed to explore its target, and it was verified in fresh biopsy tissues from 23 NPC patients and 9 patients with chronic nasopharyngitis.

**Results:**

MiR-483-5p was highly expressed in NPC tissues than in adjacent non-cancerous tissues. It was found to have a significant correlation with poor overall survival (OS) [hazard ratio (HR) = 2.89, 95% confidence interval (CI) = 1.00–8.35, *p* = 0.041] and progression-free survival (PFS) (HR = 1.95, 95%CI = 1.06–3.60, *p* = 0.029) of NPC patients. Silencing of its expression inhibited the migratory and invasive capacities of NPC cells *in vitro*. *EGR3* (early growth response 3) was identified as a direct target, and inhibiting miR-483-5p expression markedly enhanced the expression of *EGR3* at both the mRNA and protein levels. Besides, a significant decrease of *EGR3* expression was found in fresh biopsy tissues from NPC patients, in contrast to miR-483-5p expression. Furthermore, directly decreasing the expression of *EGR3* could enhance the migration and invasion of NPC cells.

**Conclusion:**

The newly identified miR-483-5p/*EGR3* pathway provides further insights into the development and metastasis of NPC and may provide a potential therapeutic target for NPC treatment in order to improve survival of NPC patients.

## Introduction

Nasopharyngeal carcinoma (NPC) is a common head and neck malignancy. Globally, the highest incident of NPC is found in southern China and southeastern Asia, where the annual incidence is about 20–50 cases per 100,000 people (1, 2). Previous studies have demonstrated that genetic susceptibility, endemic environmental factors, and Epstein–Barr virus (EBV) infection constitute the three etiological contributors to NPC (3). Although the overall survival rate is approximately 90% in patients with early clinical stage after therapy, unfortunately, most patients are diagnosed with advanced stage at their first visit, and the survival rate decreases to less than 50% (4). Recurrence and metastasis, especially high metastasis, are the major reasons for treatment failure (5). Therefore, there is a great need to fully disclose the molecular mechanism underlying the recurrence and metastasis of NPC.

MicroRNAs (miRNAs) are non-coding RNA molecules, about 19–25 nucleotides in length, negatively regulating gene expression at the posttranscriptional level through base paring with the 3′ untranslated region (3′-UTR) of the messenger RNA (mRNA) transcripts (6). In tumor biology, many studies have proven the importance of miRNAs in promoting tumor growth, metastasis, angiogenesis, and immune evasion through controlling the expressions of their target genes (7, 8). Besides, miRNAs have been developed as important biomarkers in predicting tumor prognosis (9). They provide new therapeutic targets in supporting personalized tumor therapy.

In NPC, certain amounts of valuable miRNAs have been identified in previous studies (10, 11). Some miRNAs have low expressions in tumor tissues and act as tumor suppressor genes, while some other miRNAs are highly expressed and act as oncogenes. They play important roles in the pathogenesis of NPC by regulating specific target genes that are involved in various cellular processes and pathways. The potential utility of some miRNAs as prognostic biomarkers has also been discussed (12, 13). Because of the deep influence of EBV infection, some EBV-related miRNAs have also been found to play roles in NPC (14–16). Despite great achievements having been reached, discovering more functional miRNAs is still necessary to help fully understand the mechanism of occurrence and development of NPC.

In our previous study, miR-483-5p was found to be highly expressed in plasma from NPC patients, showing its potential application in the diagnosis of NPC. Furthermore, its high expression was also validated in tumor cell lines and frozen biopsy tissues, indicating its role in causing NPC (17). In this study, the mechanism of miR-483-5p in promoting NPC was focused on. Firstly, miR-483-5p was found to have potential application in the prediction of poor prognosis. Secondly, it exerted a function in the promotion of metastasis by enhancing tumor migration and invasion. Finally, *EGR3* (early growth response 3) was identified as a functional target gene and validated by the luciferase reporter assay. Consistent with the effect of a high expression of mir-483-5p, silencing of *EGR3* could enhance the migration and invasion of NPC cell lines. The newly identified miR-483-5p/*EGR3* pathway expands our understanding of the role of mir-483-5p and may provide prognostic indicators and a novel therapeutic target for the treatment of NPC.

## Materials and Methods

### Cell Lines and Clinical Specimens

Human NPC cell lines (CNE-1 and 5-8F) were maintained in RPMI-1640 (Invitrogen, Carlsbad, CA, USA) supplemented with 10% fetal bovine serum (FBS) (Gibco, Grand Island, NY, USA). Formalin-fixed paraffin-embedded tissues of 178 primary NPC tissues and 35 adjacent non-cancerous nasopharyngeal mucosal tissues were included in the NPC tissue microarray. The detailed characteristics of the study population are presented in **Supplementary Table S1**. Besides, 32 fresh biopsy tissues from 23 NPC patients and nine patients with chronic nasopharyngitis were used for the detection of *EGR3*. The characteristics of these patients are presented in **Supplementary Table S2**. The biopsy tissues were collected at the time of diagnosis and were preserved using RNAlater (Invitrogen) in a −80°C cryogenic refrigerator before use. All samples were collected from Sun Yat-sen University Cancer Center (SYSUCC; Guangzhou, China) and reviewed by pathologists to confirm the diagnosis. The research protocols were approved by the Institutional Ethical Review Board of Sun Yat-sen University Cancer Center, and informed consent was obtained from each patient.

### RNA Extraction, Reverse Transcription, and Quantitative RT-PCR

Total RNA from cell lines and fresh biopsy tissues was extracted with TRIzol (Invitrogen) according to the manufacturer’s instructions. Complementary DNA (cDNA) was synthesized with the PrimeScript RT Reagent Kit (Takara, Tokyo, Japan). GAPDH was used as the internal control for the quantification of *EGR3*. Quantitative RT-PCR was carried out on the Roche LightCycler^®^480 96 Real-Time PCR platform, and gene expression was quantified using the 2^−ΔΔCT^ method.

### *In Situ* Hybridization

*In situ* hybridization (ISH) was conducted on the tissue microarray, which included 178 primary NPC tissues and 35 adjacent non-cancerous nasopharyngeal mucosal tissues. MiR-483-5p expression was detected by the digoxigenin (DIG)-labeled locked nucleic acid (LNA)-based probe (Qiagen, Hilden, Germany). Washing and scanning were carried out according to the manufacturer’s protocols. The sections were scored independently by two pathologists, and the staining index was generated as the product of the staining intensity (0, no staining; 1, weak, light yellow; 2, moderate, yellow brown; 3, strong, brown) and the proportion of positive cells (1, 0%–25%; 2, 26%–50%; 3, 51%–75%; 4, 76%–100%).

### Vectors and Transfection

The miRNA inhibitor, scrambled negative control (NC) oligonucleotides, and *EGR3* small interfering RNA (siRNA) were purchased from RiBoBio (Guangzhou, China). Transient transfection was performed using Lipofectamine 2000 (Invitrogen) in OPTI-MEM media according to the manufacturer’s protocol.

### Wound Healing and Invasion Assays

Cell migration was measured with a scratch wound healing assay. Transfected 5-8F cells were seeded into six-well plates, subjected to serum starvation for 24 h in serum-free media, then an artificial wound was created in the confluent cell monolayer using a 200-μl pipette tip. Images were taken at 0 and 24 h using an inverted microscope. For invasion assays, 5 × 10^4^ cells were placed into a Matrigel-coated Transwell chamber (BD Biosciences, Wokingham, UK) with an 8-μm pore size. The non-invading cells in the bottom of the chamber were fixed with 100% methanol and stained with crystal violet. The experiments were performed in triplicate.

### Luciferase Reporter Assay

The *EGR3* wild-type (Wt) and mutant (Mt) 3′-UTR sequences were synthesized and sub-cloned into the psiCHECK luciferase reporter plasmid (Promega, Madison, WI, USA). 5-8F was seeded into a 12-well plate at a density of 5 × 10^6^ and each recombinant luciferase reporter plasmid and the miR-483-5p mimic were co-transfected using Lipofectamine 2000 reagent (Invitrogen) according to the manufacturer’s instructions. The psiCHECK promoter vector was used as the control, and the pRL-SV40 Renilla luciferase vector (Promega) was used to normalize the activity of firefly luciferase. Twenty-four hours later, the luciferase activity in each well was detected using the Dual-Luciferase Reporter Assay System (Promega).

### Western Blot

Total protein was extracted from cultured cells using RIPA buffer containing phenylmethanesulfonyl fluoride (PMSF) and quantified using a bicinchoninic acid (BCA) protein assay kit (Beyotime, Haimen, China). Protein lysates were subjected to SDS-PAGE and transferred onto polyvinylidene fluoride membranes (Millipore, Billerica, MA, USA), followed by incubation first with an *EGR3* antibody (Invitrogen) and then with a secondary antibody. β-actin antibody was used as the loading control, and the bands were detected by enhanced chemiluminescence.

### Treatment and Follow-Up

All patients involved in the tissue array were treated following the routine practice of SYSUCC. Patients were followed up every 3 months during the first 2 years, semi-annually during years 3–5, and annually thereafter until death or loss to follow-up. Our primary endpoint was overall survival (OS; time from NPC diagnosis to death from any cause or censored at the date of last follow-up) and the secondary endpoint was progression-free survival (PFS; time from NPC diagnosis to the first local regional recurrence, or distant metastasis, or death from any cause, or censored at the date of last follow-up).

### Statistical Analysis

Data are presented as the mean ± SD. Student’s *t*-test was used for comparisons between groups. Univariate and multivariate logistic regressions were performed to assess the associations between the clinical characteristics and the ISH scores. For survival analyses, survival curves were depicted using the Kaplan–Meier method and compared using the log-rank test. Multivariate Cox regression analyses including age, gender, American Joint Committee on Cancer (AJCC) stage, and the ISH sores were performed. All statistical analysis was performed using R software, version 4.0.2 (http://www.r-project.org), and a two-sided *p*-value <0.05 was considered statistically significant.

## Results

### Overexpression of miR-483-5p Was Associated With Poor Prognosis in NPC Patients

**Figure 1:**
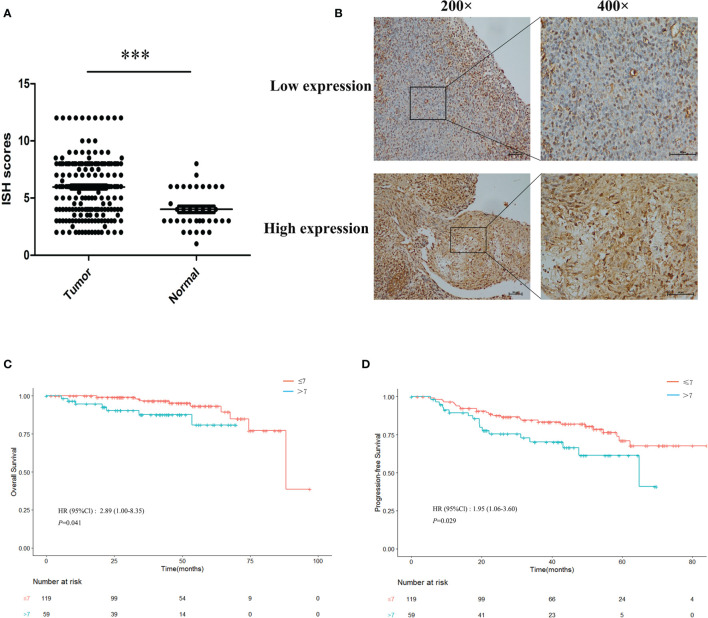
Overexpression of miR-483-5p was associated with poor prognosis in nasopharyngeal carcinoma (NPC) patients. **(A)** *In situ* hybridization (ISH) scores in NPC tissues compared with non-tumor tissues. **(B)** ISH staining of miR-483-5p in representative NPC cases with low and high miR-483-5p expressions (magnification, ×200 and ×400). **(C, D)** Survival curves for patients with NPC according to the ISH scores. ***P < 0.001.

High expressions of miR-483-5p were found in the plasma, tumor cell lines, and frozen tumor tissues from NPC patients in our previous study. To further validate its high expression, the expression level of miR-483-5p was determined using ISH in paraffin-embedded tissue microarray, which contained 178 NPC tissues and 35 non-cancerous nasopharyngeal mucosal tissues. The results showed that the ISH scores of miR-483-5p were significantly higher in tumor tissues than those in non-tumor tissues (*p* < 0.0001) (**Figure 1A**). An optimal cutoff value (COV) (ISH score = 7) for high and low miR-483-5p expressions was determined (**Figure 1B**), and 59 of the 178 (33.2%) samples were classified as high-miR-483-5p-expressing tissues (ISH scores >7). Further survival analysis established that NPC patients with a high miR-483-5p expression had significantly poorer OS (*p* = 0.041) (**Figure 1C**) and PFS (*p* = 0.029) (**Figure 1D**). Furthermore, multivariate Cox regression analysis found that miR-483-5p expression was an independent prognostic factor for PFS (HR = 1.87, 95%CI = 1.01–3.45, *p* = 0.046) (**Supplementary Table S3**). However, no significant correlations were found between miR-483-5p and any other clinical features (**Supplementary Table S4**). These results suggest that the expression level of miR-483-5p is correlated with clinical outcomes and may be a promising prognostic biomarker in NPC patients.

### MiR-483-5p Promoted NPC Cell Migration and Invasion *In Vitro*

**Figure 2:**
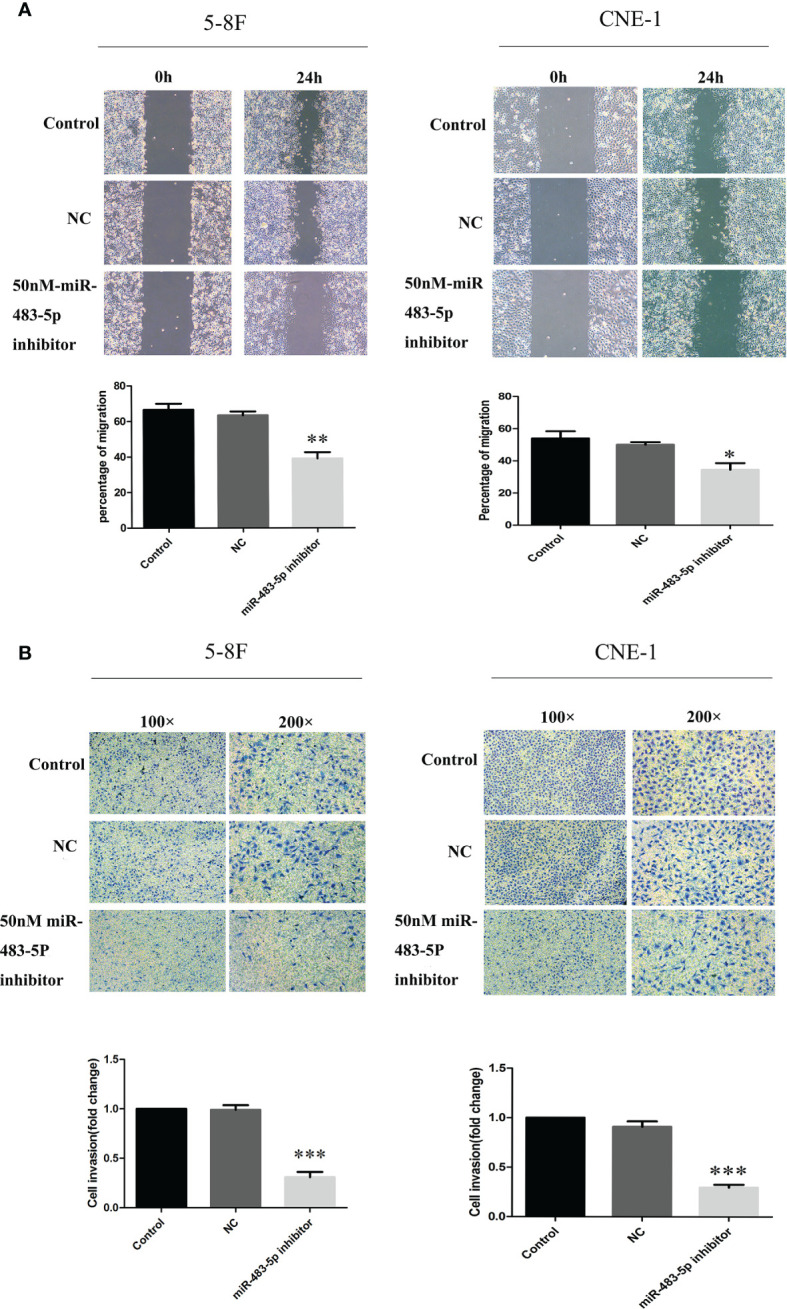
MiR-483-5p promoted the migration and invasion of nasopharyngeal carcinoma (NPC) cells *in vitro*. **(A)** Cell migration ability measured by the scratch wound healing assay. **(B)** Cell invasion ability measured by Boyden chamber assays with Matrigel. *p < 0.05, **p < 0.01, ***p < 0.001.

To determine whether ectopic expression of miR-483-5p could affect the migration and invasion abilities of NPC cells *in vitro*, wound healing and invasion assays were performed in the 5-8F and CNE-1 tumor cell lines. Because of its high expression, interfering with the miR-483-5p expression was adopted in this study. In the scratch wound healing assays, it was shown that 5-8F and CNE-1 cells transfected with the miR-483-5p inhibitor both migrated much more slowly than those transfected with the miR-Ctrl and NC (**Figure 2A**). The invasion assays showed that transfection of the miR-483-5p inhibitor significantly reduced the invasion abilities of 5-8F and CNE-1 cells (**Figure 2B**). These results suggest that the high expression of miR-483-5p promoted NPC cell migration and invasion. However, change of cell proliferation was not observed (data not shown).

### *EGR3* Was a Direct Target of miR-483-5p in 5-8F NPC Cell Line

**Figure 3:**
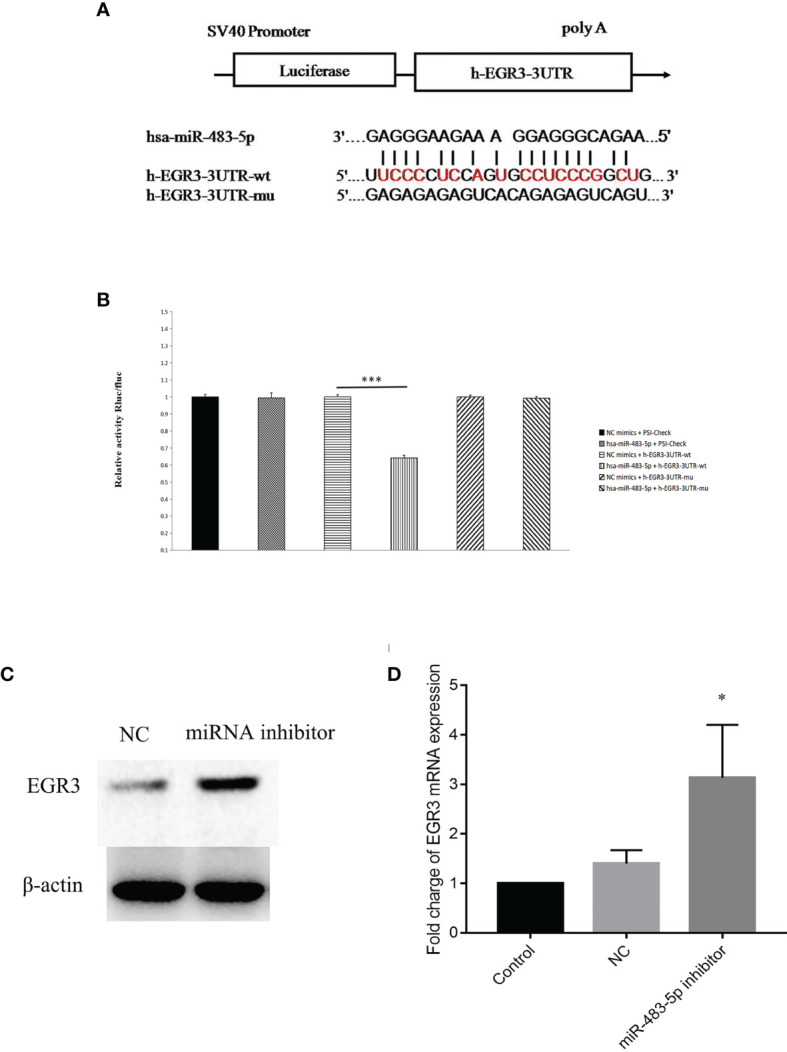
*EGR3* was a direct target of miR-483-5p in nasopharyngeal carcinoma (NPC) cell lines. **(A)** Wild-type (Wt) or mutant (Mt) target sequences of the *EGR3* mRNA 3′-UTR. **(B)** The luciferase reporter assay was performed in 5-8F cells transfected with the psiCHECK luciferase reporter plasmid containing the Wt 3′-UTR of *EGR3*, Mt 3′-UTR of *EGR3*, a miR-483-5p mimic, and the negative control. **(C)** Western blot assay of the protein level of *EGR3* in 5-8F cells after transfection with the miR-483-5p inhibitor. **(D)** Relative quantification of the mRNA expression of *EGR3* by quantitative RT-PCR in 5-8F cells after transfection with the miR-483-5p inhibitor.*p < 0.05, ***p < 0.001.

To further explore the molecular mechanism by which miR-483-5p exerts its biological function, whole-transcriptome sequencing assays were performed in the Majorbio cloud platform (https://cloud.majorbio.com). There were 101 genes with significant differences (adjusted *p* < 0.05). According to the fold change, seven genes, namely, *CCL5*, *S100A8*, *FGF21*, *S100P*, *WNT6*, *CEBPE*, and *EGR3*, were selected as the candidate targets of miR-483-5p (**Supplementary Table S5**). Afterwards, *EGR3* was predicted as a potential target gene of miR-483-5p, performed using RNA22Sites (https://cm.jefferson.edu/rna22/Interactive/). To confirm whether *EGR3* was negatively regulated by miR-483-5p, luciferase reporter vectors were constructed containing the Wt or Mt miR-483-5p target sequences of the *EGR3* 3′-UTR (**Figure 3A**). Overexpression of miR-483-5p significantly inhibited the luciferase activity of the Wt *EGR3* 3′-UTR reporter gene, but not the Mt reporter gene (**Figure 3B**). In addition, it was further found that the inhibition of miR-483-5p expression increased the expression of *EGR3* at both the protein and mRNA levels (**Figures 3C, D**, respectively) in cell lines. These results demonstrate that *EGR3* is a direct target gene of miR-483-5p.

### *EGR3* Was Decreased in NPC Clinical Specimens and Its Decrease Could Enhance NPC Cell Migration and Invasion *In Vitro*

**Figure 4:**
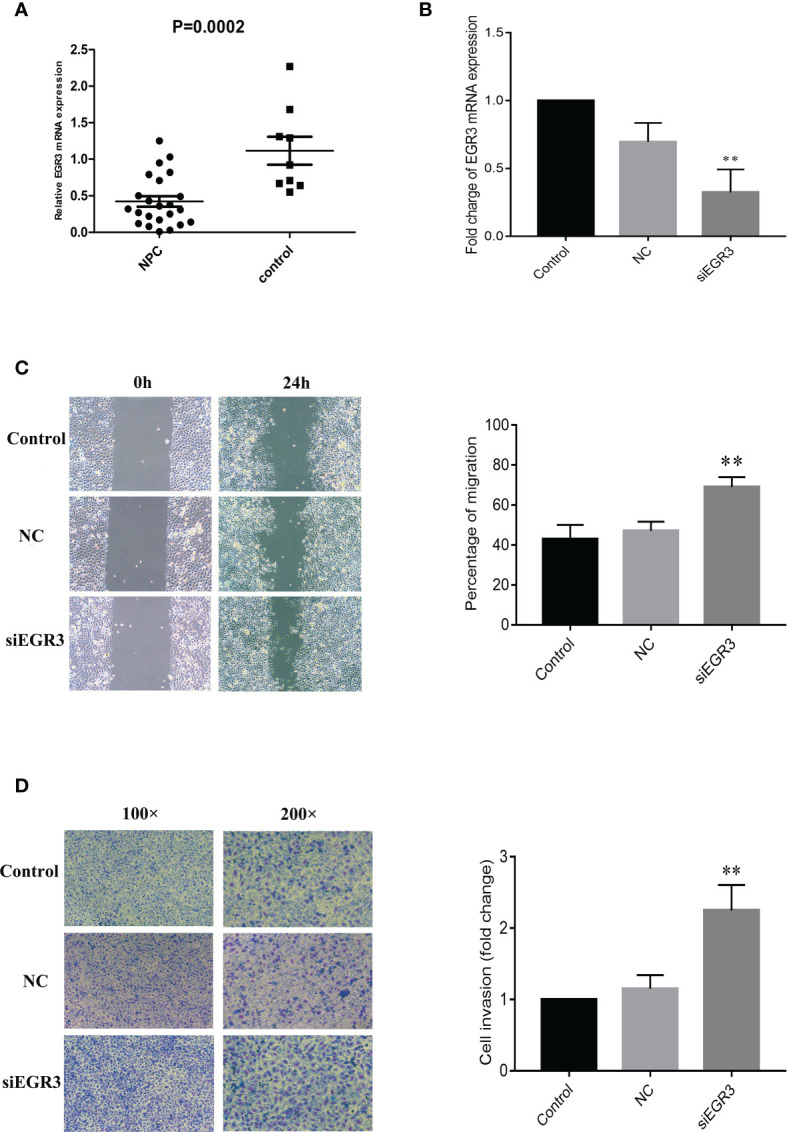
*EGR3* was decreased in nasopharyngeal carcinoma (NPC) clinical specimens, and its decrease could enhance NPC cell migration and invasion *in vitro*. **(A)** Relative expression of *EGR3* mRNA by quantitative RT-PCR in NPC tissues compared with non-tumor tissues. **(B)** Relative expression of *EGR3* mRNA by quantitative RT-PCR in 5-8F cells after transfection with siRNA. **(C)** Cell migration ability measured by the scratch wound healing assay. **(D)** Cell invasion ability measured by Boyden chamber assays with Matrigel. **p < 0.01.

The mRNA expression level of *EGR3* was further measured in fresh biopsy tissues containing 23 NPC tissues and nine non-cancerous nasopharyngeal mucosal tissues by quantitative PCR (q-PCR). As expected, the results showed that *EGR3* was significantly downregulated in tumor tissues compared with non-tumor tissues (*p* = 0.0002) (**Figure 4A**). As the inhibition of miR-483-5p has been shown to decrease the migration and invasion of NPC cells, we supposed that inhibiting the expression of *EGR3*, being a target gene of miR-483-5p, might play an opposite role in NPC. To test this hypothesis, endogenous *EGR3* in 5-8F cells was silenced by the EGR3-specific siRNA oligo (**Figure 4B**). In the wound healing assays, 5-8F cells transfected with siEGR3 migrated faster than those transfected with scrambled siRNA control and NC (**Figure 4C**). In the invasion assays, transfection with siEGR3 significantly increased the invasion ability of 5-8F cells (**Figure 4D**). Taken together, our study demonstrates that *EGR3* is a direct and functional mediator of miR-483-5p in NPC.

## Discussion

In a previous work, we found that miR-483-5p was overexpressed in the plasma, tumor cell lines, and frozen biopsy tissues from NPC patients. These results highly indicated its role in promoting NPC occurrence (17). However, there are few studies exploring its role in NPC. In this study, our results further showed that a high expression of miR-483-5p was correlated with inferior OS and PFS in NPC patients. It could promote the cell migration and invasion abilities of NPC *in vitro* by targeting *EGR3*. These findings provide new insights into the molecular functions of miR-483-5p, which could be used as a promising prognostic biomarker and a potential therapeutic target for NPC patients.

Reliable molecular biomarkers are needed for accurate prognosis. In NPC, some molecular signatures have shown potential application in prognosis prediction. For example, Liu et al. reported that a molecular signature containing five miRNAs (miR-142-3p, miR-29c, miR-26a, miR-30e, and miR-93) was significantly associated with overall, disease-free, and distant metastasis-free survival (12). Another study reported a non-overlapping four-miRNA prognostic signature, namely, miR-34c, miR-140, miR-154, and miR-449b, associated with distant metastasis in NPC (18). Considering their association with tumor metastasis, these abnormally expressed miRNAs may play roles in promoting tumor migration and invasion. Therefore, further validation studies will be essential for driving the use of these miRNAs in clinical practice for NPC. On the one hand, a high expression of miR-483-5p was not only found in the plasma, tumor cell lines, and frozen biopsy tissues from NPC patients in our study but it was also validated in the plasma from NPC patients in another study (19). On the other hand, the prognostic ability of miR-483-5p was observed in other tumors, such as in esophageal cancer (20), adrenocortical cancer (21), and hepatocellular carcinoma (22). These results provide strong evidence for its further application in prognosis prediction. As expected, miR-483-5p was further found to be associated with OS and PFS in NPC in this study (**Figure 1**).

Consistent overexpression of miR-483-5p was found in multiple types of clinical samples, such as plasma, tumor cell clines, frozen biopsy tissues, and paraffin-embedded tissues from NPC patients. These strongly suggest the potential role of miR-483-5p in NPC. Therefore, further experiments were conducted, and miR-483-5p was found to play a role in regulating tumor cell migration and invasion, but not proliferation (**Figure 2**). Two representative cell lines, a highly metastatic cell line (5-8F) and a low metastatic cell line (CNE1), were both tested. Inhibition of miR-483-5p significantly reduced the migration and invasion of NPC cell lines. Therefore, it was identified as an oncogene in NPC. However, a different function of miR-483-5p in different cancers has been shown. For example, it was found to promote cancer progression, migration, or invasion in esophageal cancer (20), gastric cancer (23), and prostate cancer (24). In contrast, it was reported to exert a function in inhibiting cell proliferation or metastasis in Wilms’ tumor (25), renal cell carcinoma (26), and glioma (27). However, its effects on the promotion of metastasis in NPC have not been reported previously.

MiRNAs exert their function by interacting with their target genes *via* base pairing to the 3′-UTR of mRNA (7, 8). Several genes such as *KCNQ1* (20), *PRM5* (24), *MKNK1* (25), and *ERK1* (27) have been identified as the target genes of mir-483-5p in esophageal cancer, prostate cancer, Wilms’ tumor, and glioma, respectively. Therefore, it seems that miR-483-5p carried out its function by regulating different target genes. Several candidate genes were identified by RNA sequencing (RNA-seq), and *EGR3* was finally validated as the direct target gene (**Figure 3**). *EGR3* is a zinc finger transcription factor and has been studied primarily in the context of neurodevelopment, autoimmunity, inflammation, and angiogenesis (28–32). Recently, several studies have shown that the expression of *EGR3* was frequently dysregulated in a variety of cancer types (33, 34). To the best of our knowledge, these observations provide the first evidence of miR-483-5p acting as a repressor of *EGR3*. The decreased expression of *EGR3* was also further validated in fresh biopsy tissues, and its decrease in tumor cell line was also found to promote the migration and invasion capacity (**Figure 4**). Besides, some other genes were found to be highly expressed, with high mir-483-5p expression, by RNA-seq. Therefore, these genes might be indirectly regulated by mir-483-5p through targeting other genes.

In conclusion, we provided the first evidence that miR-483-5p promoted the migration of NPC cell by targeting *EGR3* and may serve as a promising prognostic biomarker and therapeutic target for NPC patients. The causal link between miRNAs and tumor initiation and progression further underscores their potential utility as accurate and reliable biomarkers.

## Data Availability Statement

The original contributions presented in the study are included in the article/**Supplementary Material**. Further inquiries can be directed to the corresponding authors.

## Ethics Statement

The studies involving human participants were reviewed and approved by the Institutional Ethical Review Board of Sun Yat-sen University Cancer Center. The patients/participants provided written informed consent to participate in this study. Written informed consent was obtained from the individual(s) for the publication of any potentially identifiable images or data included in this article.

## Author Contributions

P-TY and X-HZ conceived, designed the study, and wrote the final draft. X-ZL carried out the experiments and drafted the manuscript. Y-JT carried out the experiments. J-BZ, R-WX, and P-FZ assisted in the experiments. TZ and D-WY performed data collection and analysis. All authors contributed to the article and approved the submitted version.

## Funding

This work was supported by the National Natural Science Foundation of China (grant no. 81802708), the Key Area Research and Development Program of Guangdong Province, China (grant no. 2019B110233004), the Science and Technology Planning Project of Guangdong Province, China (grant no. 2019B030316031), the Science and Technology Planning Project of Guangzhou City, China (grant nos. 201804020094 and 201904010467), the Fundamental Research Funds for the Central Universities (grant no. 19ykpy185), and the Sino-Sweden Joint Research Program (grant no. 81861138006). The funder had no role in the study design, participant recruitment, data collection, data analysis, data interpretation, or writing of the report.

## Conflict of Interest

The authors declare that the research was conducted in the absence of any commercial or financial relationships that could be construed as a potential conflict of interest.

## Publisher’s Note

All claims expressed in this article are solely those of the authors and do not necessarily represent those of their affiliated organizations, or those of the publisher, the editors and the reviewers. Any product that may be evaluated in this article, or claim that may be made by its manufacturer, is not guaranteed or endorsed by the publisher.
