# Supplementary material for: MicroRNA-483-5p Predicts Poor Prognosis and Promotes Cancer Metastasis by Targeting EGR3 in Nasopharyngeal Carcinoma
Source: Front Oncol. 2021 Oct 15;11:720835. doi: 10.3389/fonc.2021.720835 (PMC8554159; doi:10.3389/fonc.2021.720835)
Supplement: Supplementary file 1 [file Table_1.docx]

**Table S1**. Patient and tumor characteristics involved in tissue microarray

| Characteristic | Level | NPC | Normal |
| --- | --- | --- | --- |
| Number |  | 178 | 35 |
| Age | ≤44 | 93 | 16 |
|  | ＞44 | 85 | 19 |
| Gender | Male | 100 | 29 |
|  | Female | 78 | 6 |
| T stage | T1-2 | 36 |  |
|  | T3-4 | 142 |  |
| N stage | N0-1 | 88 |  |
|  | N2-3 | 90 |  |
| AJCC stage | Ⅱ | 20 |  |
|  | Ⅲ | 106 |  |
|  | Ⅳ | 52 |  |
| VCA | ≤80 | 82 |  |
|  | ＞80 | 96 |  |
| EA | ≤10 | 91 |  |
|  | ＞10 | 87 |  |
| EBV copy | ≤2000 | 98 |  |
|  | ＞2000 | 79 |  |

**Table S2**. Patient and tumor characteristics involved in fresh biopsy tissues

|  |  |  |  |
| --- | --- | --- | --- |
|  | Level | NPC | Normal |
| Number |  | 23 | 9 |
| Age | Mean(Min, Max) | 44(21,63) | 29(18,43) |
| Gender | Male | 15 | 5 |
|  | Female | 8 | 4 |
| AJCC stage | Ⅱ | 6 |  |
|  | Ⅲ | 10 |  |
|  | Ⅳ | 3 |  |
|  | Unknown^#^ | 4 |  |
| T stage | T1-2 | 8 |  |
|  | T3-4 | 11 |  |
| N stage | N0-1 | 12 |  |
|  | N2-3 | 7 |  |
| VCA | ≤80 | 4 |  |
|  | ＞80 | 15 |  |
| EA | ≤10 | 7 |  |
|  | ＞10 | 12 |  |

#The pathological staging information was evaluated by clinical doctors based on comprehensive results of magnetic resonance (MR), histopathology and clinical symptoms. Some patients were biopsy-diagnosed with NPC in our Cancer Center but subsequently moved to other hospitals for further diagnosis and treatment. For these patients, results such as MR were not collected, while only their histopathology information was obtained. The staging information of 4 patients was lost in this study.

**Table S3.** Summary of multivariate analyses of PFS and OS in NPC patients

|  | PFS |  | OS |  |
| --- | --- | --- | --- | --- |
| Variable | HR (95% CI) | *P*-Value | HR (95% CI) | *P*-Value |
| Age (>44 years vs. ≤44) | 1.11 (0.60-2.04) | 0.736 | 2.42(0.77-7.64) | 0.131 |
| Gender (male vs. female) | 1.48 (0.80-2.75) | 0.208 | 1.20(0.42-3.39) | 0.732 |
| AJCC stage ( III vs. II) | 2.14 (0.50-9.12) | 0.303 | 0.74(0.08-6.51) | 0.789 |
| AJCC stage (IV vs. II) | 3.05 (0.70-13.31) | 0.136 | 2.98(0.37-24.18) | 0.306 |
| ISH scores (>7 vs. ≤7) | 1.87 (1.01-3.45) | 0.046 | 2.75(0.92-8.18) | 0.070 |

ISH scores, In situ hybridization scores

**Table S4**. Univariate and multivariate analyses of the associations between ISH scores and clinical features

|  | level | ISH score≤7 (n=119) | ISH score>7 (n=59) | Univariate | |  | Multivariate | |
| --- | --- | --- | --- | --- | --- | --- | --- | --- |
|  |  |  |  | *OR (95%CI)* | *P* |  | *OR (95%CI)* | *P* |
| Age (％) | ≤44 | 63(52.9) | 30(50.8) | 1.00 (reference) | — |  | 1.00 (reference) | — |
|  | >44 | 56(47.1) | 29(49.2) | 1.09 (0.58-2.03) | 0.792 |  | 1.17 (0.61- 2.26) | 0.629 |
| Gender (％) | Female | 51(42.9) | 27(45.8) | 1.00 (reference) | — |  | 1.00 (reference) | — |
|  | Male | 68(57.1) | 32(54.2) | 0.89 (0.47-1.67) | 0.713 |  | 0.85 (0.44- 1.64) | 0.621 |
| T stage (％) | T1-2 | 26(21.8) | 10(16.9) | 1.00 (reference) | — |  | 1.00 (reference) | — |
|  | T3-4 | 93(78.2) | 49(83.1) | 1.37 (0.61-3.07) | 0.445 |  | 1.29 (0.57- 3.09) | 0.546 |
| N stage (％) | N0-1 | 65(54.6) | 23(39.0) | 1.00 (reference) | — |  | 1.00 (reference) | — |
|  | N2-3 | 54(45.4) | 36(61.0) | 1.88 (1.00-3.56) | 0.051 |  | 1.87 (0.97- 3.65) | 0.065 |
| VCA-IgA (％) | ≤80 | 52(43.7) | 30(50.8) | 1.00 (reference) | — |  | 1.00 (reference) | — |
|  | >80 | 67(56.3) | 29(49.2) | 0.75 (0.40-1.40) | 0.368 |  | 0.77 (0.35- 1.69) | 0.518 |
| EA-IgA (%) | ≤10 | 58(48.7) | 33(55.9) | 1.00 (reference) | — |  | 1.00 (reference) | — |
|  | >10 | 61(51.3) | 26(44.1) | 0.75 (0.40-1.40) | 0.367 |  | 0.82 (0.38- 1.77) | 0.612 |
| EBV copy (%) | ≤2000 | 67(56.8) | 31(52.5) | 1.00 (reference) | — |  | 1.00 (reference) | — |
|  | >2000 | 51(43.2) | 28(47.5) | 1.19 (0.63-2.22) | 0.593 |  | 1.05 (0.54- 2.02) | 0.891 |

**Table S5.** Candidate target gene of miR-483-5p

| mRNA | LogFC | *P*-Value |
| --- | --- | --- |
| CCL5 | 2.92 | 1.03E-11 |
| S100A8 | 2.57 | 3.58E-07 |
| FGF21 | 2.56 | 5.93E-07 |
| S100P | 2.35 | 3.30E-07 |
| WNT6 | 2.33 | 5.35E-07 |
| CEBPE | 2.24 | 5.14E-06 |
| EGR3 | -1.92 | 5.76E-06 |
